# Bioactivities and Medicinal Value of Solanesol and Its Accumulation, Extraction Technology, and Determination Methods

**DOI:** 10.3390/biom9080334

**Published:** 2019-08-02

**Authors:** Ning Yan, Yanhua Liu, Linqing Liu, Yongmei Du, Xinmin Liu, Hongbo Zhang, Zhongfeng Zhang

**Affiliations:** Tobacco Research Institute of Chinese Academy of Agricultural Sciences, Qingdao 266101, China

**Keywords:** solanesol, bioactivity, medicinal value, accumulation, extraction technology, determination methods

## Abstract

Solanesol, an aliphatic terpene alcohol composed of nine isoprene units, is mainly found in solanaceous plants. Particularly, tobacco (*Nicotiana tabacum*), belonging to the Solanaceae family, is the richest plant source of solanesol, and its leaves have been regarded as the ideal material for solanesol extraction. Since the discovery of solanesol in tobacco, significant progress has been achieved in research on solanesol’s bioactivities, medicinal value, accumulation, extraction technology, and determination methods. Solanesol possesses strong free radical absorption ability and antioxidant activity owing to the presence of several non-conjugated double bonds. Notably, solanesol’s anti-inflammatory, neuroprotective, and antimicrobial activities have been previously demonstrated. Solanesol is a key intermediate in the synthesis of coenzyme Q10, vitamin K2, and the anticancer agent synergiser *N*-solanesyl-*N*,*N*′-bis(3,4-dimethoxybenzyl) ethylenediamine. Other applications of solanesol include solanesol derivative micelles for hydrophobic drug delivery, solanesol-derived scaffolds for bioactive peptide multimerization, and solanesol-anchored DNA for mediating vesicle fusion. Solanesol accumulation in plants is influenced by genetic and environmental factors, including biotic stresses caused by pathogen infections, temperature, illumination, and agronomic measures. Seven extraction technologies and seven determination methods of solanesol are also systematically summarized in the present review. This review can serve as a reference for solanesol’s comprehensive application.

## 1. Introduction

Solanesol, an aliphatic terpene alcohol composed of nine isoprene units, is mainly found in Solanaceae plants such as tobacco (*Nicotiana tabacum*), tomato (*Solanum lycopersicum*), and potato (*Solanum tuberosum*) [1,2,3,4,5]. As the chemical synthesis of solanesol is difficult due to its long carbon chains [6], solanesol is mainly obtained through extraction from solanaceous plants [1,2,3,4,7]. To the best of our knowledge, tobacco is the richest plant source of solanesol; among the various organs of tobacco plants, leaves possess the highest content of solanesol, followed by stems and roots [1,2,8]. Therefore, tobacco leaves have been regarded as the ideal material for solanesol extraction. Solanesol accumulation in the leaves of plants is affected by genetic and environmental factors [1,9,10]. The solanesol content of tobacco leaves are jointly determined by the major genes and polygenes, with the major genes being dominant, and is also influenced by environmental factors [10]. Pathogen infections, temperature, illumination, and agronomic measures influence solanesol accumulation in tobacco plants [1,2,3,4]. Notably, solanesol content in tobacco leaves is the main limiting factor for its extraction.

**Figure 1:**
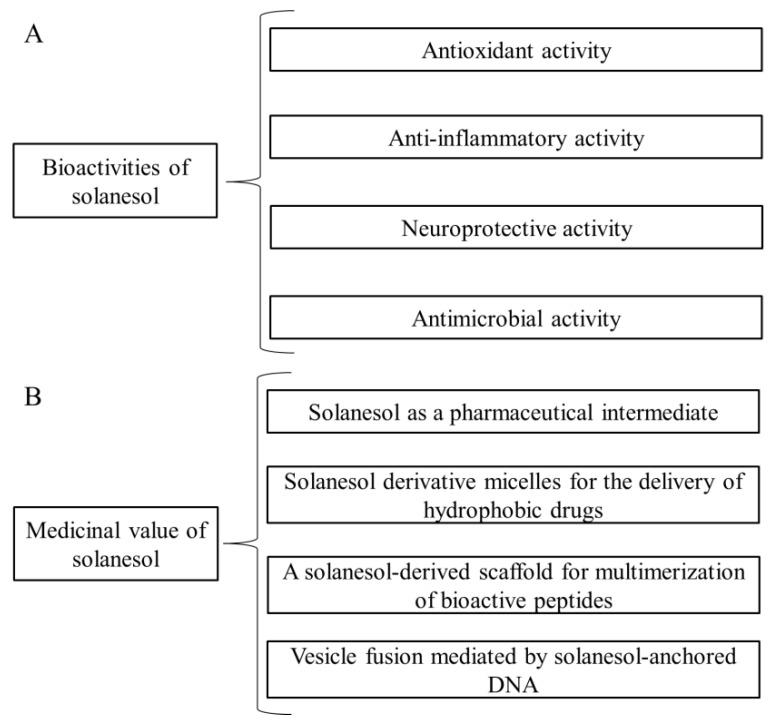
Bioactivities (**A**) and medicinal value (**B**) of solanesol.

Since the discovery of solanesol in tobacco by Rowland et al. [11], significant progress has been achieved in research on its bioactivities, medicinal value, biosynthesis, extraction technology and determination methods [1,2,3]. In a previous review, we provided a detailed summary of the physiological functions of solanesol in plants as well as the biosynthetic pathway and key enzymes of solanesol biosynthesis [3]. Other studies have proven that solanesol possesses antioxidant [12,13], anti-inflammatory [14], neuroprotective [15], and antimicrobial [16] activities (Figure 1). Notably, solanesol serves as an intermediate in the synthesis of coenzyme Q10, vitamin K2, and the anticancer agent synergiser *N*-solanesyl-*N*,*N*′-bis(3,4-dimethoxybenzyl) ethylenediamine (SDB) [1,8], and solanesol derivative micelles have been used in the delivery of hydrophobic drugs [17,18]. An economic crop widely planted in the world, tobacco has marked medical value because of its rich content of solanesol and other beneficial active ingredients [1,2,3,4,5]. Moreover, the extraction technology and determination methods of solanesol are the basis for further research, development, and application [5,11]. Therefore, this review provides a detailed summary of solanesol’s bioactivities, medicinal value, accumulation, extraction technology, and determination methods to serve as a reference for its comprehensive application.

## 2. Bioactivities of Solanesol

### 2.1. Antioxidant Activity

Solanesol possesses strong free radical absorption ability due to the presence of several non-conjugated double bonds [1]. In addition, this compound has excellent antioxidant activity, with its scavenging activities for superoxide anion and hydroxyl radicals being comparable to those of Trolox [19]. Huang et al. [12] performed ethanol-modified supercritical CO_2_ extraction of crude solanesol from tobacco leaves and observed that crude solanesol exhibited good anti-free radical activity (2,2 diphenyl-1-picrylhydrazyl radical-scavenging activity). Meanwhile, it was found that the extract’s anti-free radical activity correlated very well with the crude solanesol yield, resulting in a model with a high coefficient of determination (R^2^ = 0.946). Furthermore, solanesol can effectively absorb ultraviolet radiation and inhibit tyrosinase, which is a key enzyme in melanin synthesis and closely linked to human pigmentation disorders [20]. Therefore, solanesol may potentially be used in the inhibition of skin ageing and age spots formation. In a study by Yao et al. [13], it was found that solanesol protected human hepatic L02 cells from ethanol-induced oxidative injury via upregulation of haem oxygenase-1 (HO-1), an inducible enzyme with major antioxidant and anti-inflammatory functions [21], and heat shock protein 70, which plays a key cytoprotective role in protein homeostasis and participates in multiple cellular signaling events [22]. A recent study by Banožić et al. [23] showed that solanesol and phenolics are the main bioactive compounds in the extracts of different types of tobacco industry wastes (scrap, dust, and midrib). Furthermore, noticeable differences in bioactive compound content and antioxidant activity are found in extracts related to ultrasound-assisted extraction conditions and type of tobacco waste.

### 2.2. Anti-Inflammatory Activity

Solanesol has been extracted from tobacco scrap and tested for its topical anti-inflammatory activity against carrageenan-induced hind paw oedema [24]; the results revealed that the developed solanesol herbal gel formulation demonstrated comparable topical anti-inflammatory properties. Yao et al. [14] examined the anti-inflammatory effect of solanesol and elucidated the underlying mechanisms. First, solanesol induced HO-1 (an enzyme that plays an important role in cytoprotection against oxidative stress and inflammation [21]) expression both at the mRNA and protein levels, resulting in increased HO-1 activity in RAW264.7 cells. Second, solanesol treatment activated both p38 and Akt, also treatments with SB203580 (a p38 kinase inhibitor), LY294002 (an Akt inhibitor), specific p38 siRNA, and Akt siRNA suppressed the solanesol-induced activation of Nrf2, resulting in a decrease in HO-1 expression. Thus, it is quite plausible that HO-1 induction by solanesol could trigger anti-inflammatory pathways including limiting of lipopolysaccharide-stimulated cytokine production through autophagic signaling via p38 and Akt [14]. Recently, Zhang et al. [25] found that intragastric administration of solanesol alleviated systemic oxidative stress and reduced the expression of pro-inflammatory cytokines in rats with ligature-induced periodontitis. The results of this study can serve as a reference for clinical applications of solanesol and the use of solanesol for the improvement of periodontal fillings. Another study by Gao et al. [26] showed that solanesol had strong static quenching effects on bovine serum albumin. Through further analysis, it was found that hydrophobic interactions played a key role in the binding of solanesol with bovine serum albumin, and solanesol could be inserted into bovine serum albumin at tryptophan 212. The results of this study may serve as a reference for the synthesis and clinical use of solanesol-based drugs.

### 2.3. Neuroprotective Activity

Huntington’s disease (HD) is a hereditary autosomal dominant disorder of the central nervous system [27]. Despite various advancements in HD research, no treatment strategy has been found to be effective for curing or combating all the symptoms associated with the disease. Hence, solanesol is a phytochemical that can influence different pathological pathways and facilitate the amelioration of all symptoms and can be considered potent for the management of HD [15]. Mehan et al. [28] evaluated the solanesol-mediated coenzyme-Q10 restoration to ameliorate 3-nitropropionic-induced behavioral, biochemical, and histological changes, which resemble HD-like symptoms in men. Solanesol treatment significantly improved motor performance and cognitive behavior-related tasks, restored histopathological changes, improved mitochondrial complexes such as coenzyme-Q10 enzyme activity, and attenuated inflammatory and oxidative damage to the rat brain. Based on the observed data, it was proposed that solanesol may be utilized as a therapeutic agent/adjuvant to manage oxidative stress-mediated neurodegeneration.

### 2.4. Antimicrobial Activity

Solanesol has good antibacterial and antiviral activities [1,16]. Chen et al. [29] showed that solanesol has significant inhibitory effects against *Escherichia coli*, *Mycobacterium phlei*, *Pseudomonas aeruginosa*, and *Staphylococcus aureus*, but poor inhibitory effects against *Bacillus circulans* and *Bacillus subtilis*. Based on the antifungal activity of pure and mixed solanesol, chlorogenic acid, rutin, and nicotine and on the correlation between the content of the main antifungal metabolites of tobacco extract and their antifungal activity, it was found that cembranoids may be the main antifungal substance [30]. Therefore, the antifungal activity of solanesol was not found to be significant. Notably, solanesol is considered a key effective substance in tobacco mosaic virus (TMV)-resistant tobacco varieties [31].

## 3. Medicinal Value of Solanesol

### 3.1. Solanesol as a Pharmaceutical Intermediate

**Figure 2:**
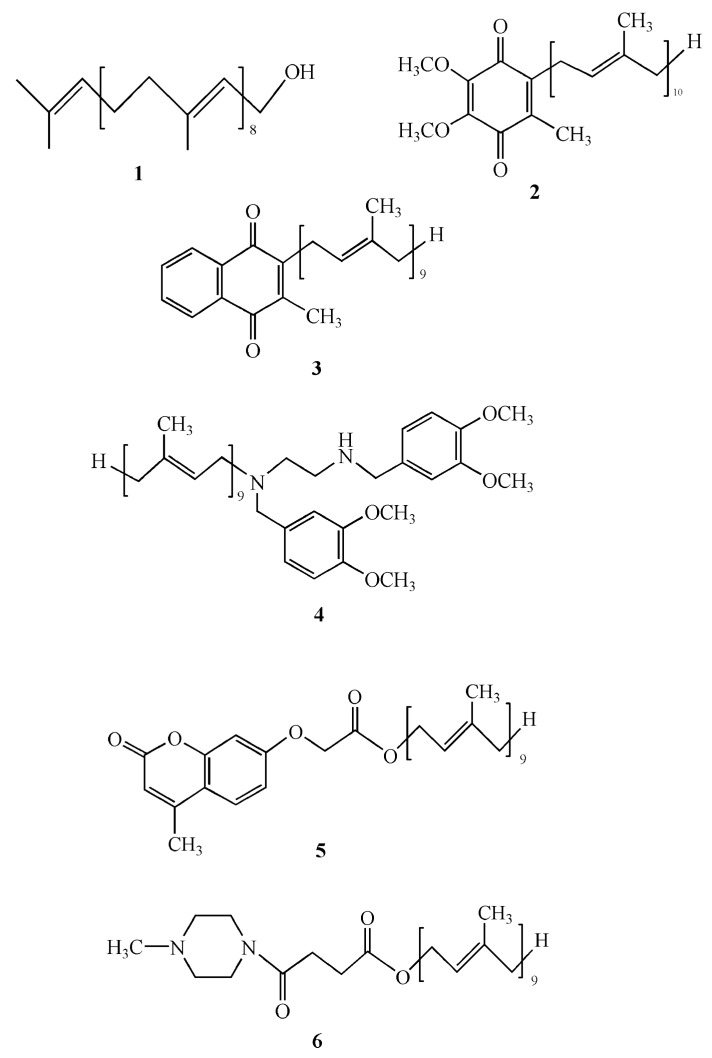
Chemical structure of solanesol (**1**) and its key derivatives (**2**–**6**).

Solanesol (**1**) serves as a key pharmaceutical intermediate in the synthesis of ubiquinone drugs, including coenzyme Q10 (**2**) and vitamin K2 (**3**), and the anticancer agent synergizer SDB (**4**) (Figure 2) [1,3,8]. Coenzyme Q10, a lipid-soluble quinone widely distributed within organisms, plays an essential role in the mitochondrial respiratory chain. It participates in oxidative phosphorylation, ATP synthesis, and acts as an activator of cellular metabolism and cellular respiration [32]. Notably, coenzyme Q10 possesses several physiological functions, including high antioxidant activity, enhancement of human immunity, strengthening of cardiac power, improvement of brain function, and regulation of blood lipids. Therefore, it can be used for the treatment of diseases such as neurodegenerative diseases, hypertension, and cardiovascular diseases [33] and as a dietary supplement for patients with type 2 diabetes [34]. Vitamin K2, a lipid-soluble vitamin, is closely linked to bone metabolism and has positive effects in the prevention and treatment of osteoporosis [35]. In addition, vitamin K2 promotes blood clotting and inhibits vascular calcification [36]. SDB and its derivatives have been found to significantly reverse the drug resistance of the paclitaxel-resistant KK47/TX30 cell line, with the mechanism of action being related to their inhibitory effects against P-glycoprotein-mediated multidrug resistance [37,38]. Solanesol can also be utilized for the modification of traditional anticancer drugs to enhance the tumor suppression effects and reduce the toxicity of anticancer drugs. Xiao et al. [39] synthesized 12 new diacid solanesyl 5-fluorouracil ester derivatives and successfully screened these target compounds with good antitumor activity and low toxicity. Notably, the molecular dynamics simulation analysis of solanesol docked with focal adhesive kinase has also proven that solanesol acts as an anticancer agent [40]. A series of compounds have been synthesized from solanesol, and these compounds have been evaluated for antioxidant, angiogenesis, and wound-healing activity [41]. Of these, two compounds (**5** and **6**) exhibit better wound-healing activity than that exhibited by 3,3-dimethylacryl shikonin, indicating that these two solanesol derivatives can be further developed as wound-healing agents.

### 3.2. Solanesol Derivative Micelles for the Delivery of Hydrophobic Drugs

Micelles of amphiphilic block copolymers have been used for delivering insoluble drugs because they have a hydrophobic core, which can encapsulate poorly water-soluble drugs and improve the pharmacokinetic and pharmacodynamic properties of the drug. To improve the bioavailability and tumor targeting capability of the hydrophobic drug, Qin et al. [17,18] synthesized amphiphilic solanesol derivatives by esterification using solanesol as a raw material for the delivery of coenzyme Q10 and the small-molecule anticancer drug doxorubicin (DOX). As coenzyme Q10 exhibits extremely low solubility in an aqueous medium as well as poor oral bioavailability, solanesyl poly (ethylene glycol) succinate (SPGS) and coenzyme Q10 were formulated as coenzyme Q10-SPGS micelles with a high content of coenzyme Q10 to improve the bioavailability of coenzyme Q10 in rat [18]. In vivo experiments demonstrated that compared to that of the coarse suspensions of coenzyme Q10, there was a three-fold enhancement of oral bioavailability for coenzyme Q10-loaded SPGS micelles depending on the varied molecular weight of SPGS. Qin et al. [17] prepared an amphiphilic redox-responsive conjugate based on mPEGylated solanesol, solanesyl poly (ethylene glycol) dithiodipropionate (SPDP), along with its inert counterpart SPGS, which self-assembled in an aqueous solution to form redox-responsive micelles, which were used as efficient drug carriers for DOX and acted as synergistic agents for cancer therapy.

### 3.3. Other Values of Solanesol

#### 3.3.1. A Solanesol-Derived Scaffold for Multimerization of Bioactive Peptides

A flexible molecular scaffold bearing varying numbers of terminal alkyne groups was synthesized from solanesol in five steps [42]. R(CO)-MSH(4)-NH_2_ ligands, which have a relatively low affinity for binding at the human melanocortin 4 receptor (hMC4R), were prepared by solid-phase synthesis and were N-terminally acylated with 6-azidohexanoic acid. Multiple copies of the azide N_3_(CH_2_)_5_(CO)-MSH(4)-NH_2_ were attached to the alkyne-bearing, solanesol-derived molecular scaffold via a copper(I)-catalyzed azide-alkyne cycloaddition (CuAAC) reaction. In a competitive binding assay with an Eu-labelled probe based on the superpotent ligand NDP-α-MSH, the monovalent and multivalent constructs appear to bind to hMC4R as monovalent species. In a similar assay with an Eu-labelled probe based on MSH(4), modest increases in binding potency with an increased MSH(4) content per scaffold were observed.

#### 3.3.2. Vesicle Fusion Mediated by Solanesol-Anchored DNA

Fusion between two lipid bilayers is one of the central processes in cell biology, playing a key role in endocytosis, exocytosis, and vesicle transport. Flavier and Boxer [43] tested the hypothesis that the length of the membrane anchor may affect the outcome by comparing single leaflet-spanning DNA-lipid-mediated vesicle fusion with fusion mediated by DNA anchored by solanesol, a C45 isoprenoid of sufficient length to span the bilayer. When the solanesol anchor was present on the incoming vesicles, target membrane, or both, ~2–3 times as much full fusion was observed as in the DNA-lipid mediated system, as measured by lipid mixing or content transfer, indicating that a transmembrane anchor increases the efficiency of full fusion.

## 4. Factors Associated with Solanesol Accumulation in Plants

### 4.1. Genetic Factors

The accumulation of solanesol in plants is influenced by genetic and environmental factors [1,9], with genetic factors exerting a considerable influence on solanesol accumulation. To identify solanesol-rich tobacco varieties, our research team measured the solanesol content in leaves of 93 tobacco germplasms and found that the solanesol content in different tobacco varieties ranged from 1.78% to 3.60% of dry matter [1]. Campbell et al. [9] characterized the genetic variation in leaf solanesol content in a biparental, segregating diploid potato population. They demonstrated that the solanesol content in those potato leaves was genetically controlled and identified several quantitative trait loci associated with leaf solanesol content. Xiang et al. [10] measured the solanesol content in 168 Chinese flue-cured tobacco germplasm resources, planted in four geographical regions of China in 2014 and 2015. Their results indicate that the solanesol content of flue-cured tobacco ranged from 0.70% to 4.13%. To elucidate the influence of key enzyme genes for terpenoid metabolism on solanesol synthesis in tobacco, Gai et al. [44] measured the solanesol content in roots, stems, and leaves at different developmental stages and the expression of key enzyme genes for terpenoid metabolism of the tobacco cultivar Honghuadajinyuan with a high solanesol content and cultivar Zhongyan90 with a low solanesol content. Results indicate that the key enzyme genes for terpenoid metabolism in both Honghuadajinyuan and Zhongyan90 may jointly regulate the synthesis and accumulation of solanesol through synergistic effects. To explore the hereditary patterns of genes related to solanesol content in tobacco leaves, Xiang et al. [45] formulated hybrid combinations using the tobacco cultivar Maryland 609, which has a low solanesol content, as the maternal plant and the cultivar K326, which has a high solanesol content, as the paternal plant. They produced populations of four generations P_1_, P_2_, F_1_, and F_2_. Results of a genetic analysis of the populations indicate that the solanesol content of the Maryland 609 × K326 combination was controlled by two major genes with equal additive effects plus polygenes with additive-dominant effects. The heritability of the major genes was estimated to be 33.61% and 53.15%, respectively, and the corresponding heritability of the polygenes was estimated to be 30.01% and 13.64%, respectively; environmental variation was within 33.21%–36.28%. Therefore, the solanesol content of tobacco leaves was jointly determined by the major genes and polygenes, with the major genes being dominant, and was influenced by environmental factors as well.

### 4.2. Environmental Factors

Solanesol accumulation in plants is a complex, dynamic process influenced by genetic factors, environmental factors, and the developmental stages of plants [1,9]. The solanesol content in the roots, stems, and leaves of tobacco is relatively low from the seedling stage to the rosette stage. Slow solanesol accumulation occurs in the various organs of tobacco plants from the flourishing stage to the budding stage. The rate of solanesol accumulation reaches a maximum from the flowering stage to the leaf maturation stage, and solanesol content is at its highest in the various organs during the leaf maturation stage [44]. Solanesol plays a key role in the response of tobacco to biotic stresses caused by the TMV or *Pseudomonas syringae* pv. *tabaci* [31] and environmental factors such as moderately high temperature [4]. In a previous study, it was found that upon TMV or *P. syringae* pv. *tabaci* infection, the increase in solanesol content was not significant in infected plants. On the other hand, resistant plants show a significant increase in solanesol content, which indicates that solanesol plays a crucial role in the response of tobacco to biotic stresses such as TMV or *P. syringae* pv. *tabaci* infections [31]. Exposure to moderately high temperatures (day/night temperature, 30 °C/24 °C) resulted in a significant increase in the net photosynthetic rate and solanesol content in tobacco leaves [4]. Results of RNA sequencing showed that exposure to moderately high temperatures results in the upregulation of 492 genes and downregulation of 1440 genes, and the 122 transcription factors of differentially expressed genes could be divided into 22 families. Notably, upregulation of *N. tabacum* 3-hydroxy-3-methylglutaryl-CoA reductase, 1-deoxy-d-xylulose 5-phosphate reductoisomerase, geranylgeranyl diphosphate synthase, and solanesyl diphosphate synthase genes promoted rapid solanesol accumulation in tobacco leaves at moderately high temperatures [4]. Similarly, another study showed that the solanesol content of potato leaves that were exposed to moderately high temperatures (day/night temperature, 30 °C/20 °C) for 1 week was enhanced by up to six-fold compared with that of potato leaves grown at normal temperatures (day/night temperature, 22 °C/16 °C), which indicates that solanesol plays a key role in the response of potato to moderately high temperatures [9]. Besides temperature, illumination also influences solanesol accumulation in tobacco, as shown by a decrease in the solanesol content of tobacco leaves under shaded conditions [46]. In addition, the transient over-expression of genes from the methylerythritol 4-phosphate (MEP) and mevalonic acid (MVA) pathways, either singly or in combination, resulted in enhanced accumulation of solanesol in the leaves of *Nicotiana benthamiana*, providing insights for genetically engineering the pathway [9]. Other studies have shown that agronomic measures influence solanesol accumulation. For instance, tobacco topping led to a marked increase in solanesol content at the top stalk position of tobacco plants [47], and appropriate dryness from topping to the maturation stage can promote solanesol accumulation in burley tobacco leaves [48]. Therefore, biotic stresses such as pathogen infections and environmental factors such as temperature, illumination, and agronomic measures influence solanesol accumulation in plants.

## 5. Extraction Technology of Solanesol

**Figure 3:**
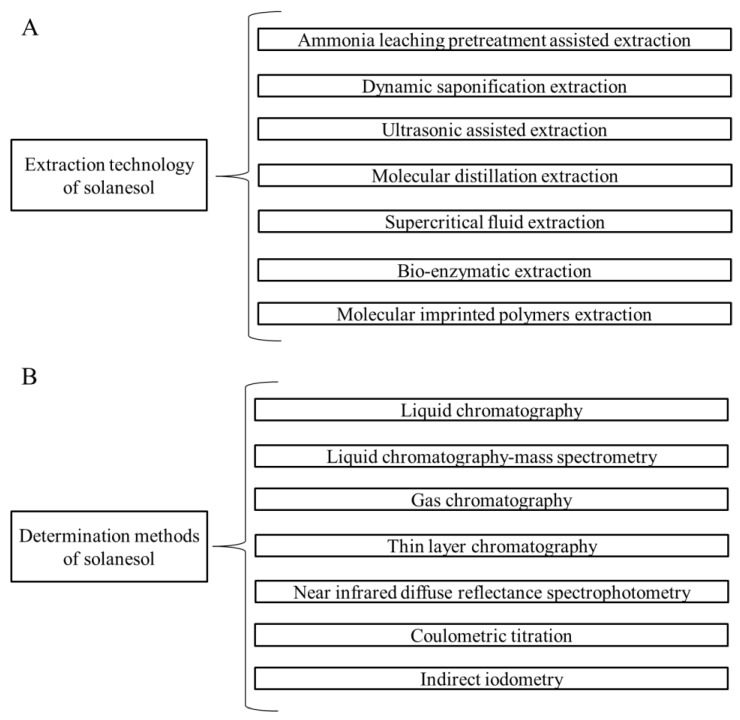
Extraction technology (**A**) and determination methods (**B**) of solanesol.

Extraction technology of solanesol primarily includes ammonia leaching pretreatment-assisted extraction, dynamic saponification extraction, ultrasonic assisted extraction, molecular distillation extraction, supercritical fluid extraction, bio-enzymatic extraction, and molecular imprinted polymers (MIPs) extraction (Figure 3).

### 5.1. Ammonia Leaching Pretreatment Assisted Extraction

Ammonia leaching pretreatment utilizes hexane as a solvent to extract the solanesol in tobacco. Ammonia leaching pretreatment is performed prior to extraction to enhance the effectiveness of the extraction. Sun et al. [49] showed that ammonia leaching pretreatment not only destroyed the cell wall structure of tobacco leaves, promoted the release of solanesol, hydrolyzed and saponified solanesol ester into free solanesol, but also promoted the dissolution of certain impurities and enhanced the extraction effectiveness for solanesol. Following ammonia leaching pretreatment of raw tobacco leaves, the equilibrium time of extraction was shortened from 3 to 1.5 h, the extraction yield of solanesol was increased from 89.24% ± 1.54% to 104.63% ± 2.44%, and the purity of the extract was increased from 16.72% ± 0.60% to 21.03% ± 0.60%.

### 5.2. Dynamic Saponification Extraction

Dynamic saponification extraction utilizes ethanol and solvent naphtha as solvents to extract the solanesol in tobacco via saponification. Zhao et al. [50] showed that the optimal parameters for dynamic saponification extraction were sodium hydroxide as saponification reagent; no. 6 solvent naphtha (Petrochina Daqing Petrochemical Co., Ltd., Daqing, Heilongjiang, China) as solvent for the crude extraction of tobacco leaves; sodium hydroxide solvent in 80% ethanol by volume fraction; the mass ratio of sodium hydroxide to tobacco crude extract was 1:4; and the saponification reaction time was 2.5 h. Under these optimized conditions, the yield of solanesol after dynamic saponification treatment increased by 9.30% compared with conventional saponification treatments; the mass fraction of solanesol in tobacco crude extract increased by 2.94% compared with conventional saponification treatment. Meanwhile, dynamic saponification extraction reduced the number of experimental procedures and lowered the consumption of extractants, thereby enabling the rapid and efficient extraction of solanesol.

### 5.3. Ultrasonic Assisted Extraction

Compared with the organic solvent extraction methods, ultrasonic assisted extraction has advantages such as rapid extraction, high extraction efficiency, low usage of solvent, and simple operation procedures. Zhang et al. [51] identified the optimal conditions for ultrasonic assisted extraction of solanesol from tobacco using an orthogonal experimental design: acetone was used as the extraction solvent. The raw material-to-liquid ratio was 1:17.5 (g/mL), the temperature was 60 °C, the extraction time was 2 h, and the ultrasonic power was 160 W. In particular, the extraction time was the main factor affecting the extraction yield of solanesol; the extraction yield of solanesol using ultrasonic assisted extraction could reach 94.70%.

### 5.4. Molecular Distillation Extraction

Molecular distillation is a continuous distillation process performed under high-vacuum conditions. It is suitable for mixtures with a high boiling point and high viscosity, as well as in heat-sensitive and biologically active mixtures. Qian and Zhang [52] utilized wiped film molecular distillation units to study the refining of solanesol. They found that the crude solanesol did not require pretreatment, such as decolorization, and high purity solanesol was obtained after merely two molecular distillations. Specifically, the optimal conditions for solanesol extraction using molecular distillation were feed rate of 350–400 mL/h, feed temperature of 100 °C, distillation temperature of 200–220 °C, evaporation pressure of 10 Pa, and agitation speed of 300–400 r/min. The final purity and yield of solanesol reached 97.60% and 77.10%, respectively. Their study demonstrates that it is feasible to refine crude solanesol using molecular distillation units.

### 5.5. Supercritical Fluid Extraction

Supercritical fluid extraction is a novel refining technology that utilizes a supercritical fluid as an extractant to extract and separate active components from a fluid or liquid. This method requires relatively low extraction temperature, which not only protects the effective components from being damaged, but also retains their original biological activities. In addition, this method has a short extraction time, and displays exceptionally high solubility for fat-soluble substances. Wei et al. [53] found that the experimental conditions for the extraction of solanesol using supercritical CO_2_ were extraction pressure of 20 MPa, extraction temperature of 55 °C, extraction time of 1.5 h, and 95% ethanol as modifier. These conditions resulted in crude solanesol with a mass fraction of 30%. Then, the crude solanesol was subjected to silica gel column chromatography, subsequently being separated and eluted with petroleum ether-ether-ethyl acetate-acetone (15:5:3:1, V/V/V/V). The relevant fractions were collected and recrystallized to obtain solanesol product with a mass fraction of greater than 98%. Wang and Gu [54] found that the extraction rate of solanesol is higher than that of tobacco extract, indicating that a shorter extraction time results in higher selectivity of the molecule. Furthermore, pretreatment with hexane:95% ethanol at a 4:6 volumetric ratio was performed to improve the selectivity of solanesol extraction.

### 5.6. Bio-Enzymatic Extraction

Bio-enzymatic extraction utilizes the highly specific and highly efficient characteristics of biological enzymes, in which a corresponding biological enzyme is selected to degrade the plant cell wall to completely release the effective component, thereby realizing the extraction of the effective component. Wang et al. [55] utilized combined cellulase and ligninase to catalyze the degradation of waste tobacco leaves, and investigated the clean and efficient method for enzymatic cell-wall breaking and the conditions for leaching solanesol. They found that the optimal conditions for bio-enzymatic extraction of solanesol were a combination of enzymes with a cellulase:ligninase ratio of a 15:1 (U/U) and a water medium with volume five times to that of tobacco. When the combined enzyme activity was 175 U/g, the water bath temperature was 40 °C, the pH was 6, and the tobacco leaves had been catalyszd by enzymes for 8 h, the concentration of solanesol in the solution could reach 0.33 g/L. Under these conditions, the average extraction yield of solanesol could reach 96.53%, which was 1.68 times of that by the chemical reflux extraction method.

### 5.7. Molecular Imprinted Polymers Extraction

MIPs of solanesol were synthesized through solution polymerization using solanesol as the template molecule, *N*-vinylpyrrolidone and acrylic acid as functional monomers, ethyleneglycol dimethacrylate as cross-linker, and azodiisobutyronitrile as initiator [56]. The selectivity of prepared MIP was evaluated, and samples with a highly selective separation degree of 3.5 for solanesol were prepared. Scatchard analysis revealed that heterogeneous binding sites were formed in solanesol-MIP [56]. Ma et al. [57] synthesized spherical MIP particles for solanesol within a size range of 250–350 μm (d (0.5) = 320 μm) using suspension polymerization, with an imprinting factor of 3.9. MIP particles (5.5 g) were packed in a common Teflon column as the stationary phase with methanol and methanol/acetic acid solution (80/20, *V*/*V*) as sample solution and elution solvent, respectively, and loading at 4 mL/min and eluting 8 mL/min. Under optimal chromatographic conditions, the adsorption capacity of the MIP-Flash column was determined as 107.3 μmol/g, and in each process, 370.8 mg of purified solanesol (98.4%) were obtained from the extract (20 mM, 40 mL) of tobacco leaves (14.7 g), and the yield of solanesol was 2.5% of the dry weight of tobacco leaves. Surface MIPs of solanesol were prepared using reversed phase suspension polymerization and modified titanium dioxide as carrier, and operation conditions were investigated and optimized [58]. Adsorption kinetics results indicate that the adsorption of surface MIPs of solanesol to solanesol was a pseudo-second order process, and the adsorption at the early and late stages was controlled by homogeneous particle diffusion and adsorption reaction process, respectively.

## 6. Determination Methods of Solanesol

Determination methods of solanesol primarily include liquid chromatography (LC), liquid chromatography-mass spectrometry (LC-MS), gas chromatography (GC), thin layer chromatography (TLC), near infrared diffuse reflectance spectrophotometry (NIRDRS), coulometric titration, and indirect iodometry (Figure 3).

### 6.1. Liquid Chromatography

Solanesol is a compound with high molecular weight and high boiling point. Thus, liquid chromatography is the most frequently used method for the determination of solanesol. Currently, liquid phase detectors for solanesol determination mainly include ultraviolet detectors (UV), refractive index detectors (RID), and evaporative light scattering detectors (ELSD).

#### 6.1.1. Ultraviolet Detector

Liquid chromatography-ultraviolet detector (LC-UV) is currently the most frequently used detector during the determination of solanesol. The determination of free solanesol and total solanesol using LC-UV has been widely adopted by both domestic and foreign researchers. However, the pretreatment method for determining total solanesol is more complicated and requires saponification reaction to transform bound solanesol into free solanesol [59]. Both reversed phase high performance liquid chromatography (RP-HPLC) and normal phase high performance liquid chromatography (NP-HPLC) can be used for the determination of solanesol, but most studies tend to use RP-HPLC [60,61]. The mobile phase mainly utilizes either methanol, acetonitrile, isopropanol, hexane, or combinations thereof. The optimal UV absorption wavelength for different solanesol determination methods is slightly different, but the wavelength is always ranged between 205 and 215 nm. Most studies have selected the external standard method for calibration during the quantitative analysis of solanesol [62].

#### 6.1.2. Refractive Index Detector

As a general-purpose detector, RID can be used for all substances, including for the determination of solanesol. Zhao et al. [63] utilized a mixture of hexane-isopropyl ether-ethyl acetate as mobile phase, a flow rate of 1.0 mL/min, a Zorbax-SIL (250 × 4.6 mm i.d.) chromatography column for separation and determined the total solanesol content in tobacco leaf extracts using RID. Such a method yielded satisfactory separation and recovery. Zhang et al. [64] selected the ODS (250 × 4.6 mm i.d.) chromatography column, methanol-isopropanol (50:50, *V*/*V*) as mobile phase, and a flow rate of 1.0 mL/min, to determine the total content of solanesol in tobacco leaves using RID. Their method shows good precision and accuracy when the solanesol injection volume is 1–100 μg.

#### 6.1.3. Evaporative Light Scattering Detector

Compared with RID, ELSD exhibits advantages such as high sensitivity, low sensitivity to temperatures, stable baseline, and the feasibility to perform gradient elution. Li and Qian [65] were the first to determine the free solanesol content in tobacco leaves using liquid chromatography-evaporative light scattering detector (LC-ELSD): the chromatography column used was Discovery™ C_18_ (250 × 4.6 mm i.d.), the mobile phase was methanol-acetonitrile-tetrahydrofuran (39:24:9.5, V/V/V), and the flow rate was 1.0 mL/min. Zhou and Liu [66] utilized acetonitrile-isopropanol (60:40, V/V) as mobile phase, the C_18_ (150 × 4.6 mm, 5 μm) chromatography column for separation, and used ELSD to detect free solanesol in tobacco leaves. This method provided satisfactory results, with a precision of 0.68%–5.16% and a recovery of 97.80%–101.92%.

### 6.2. Liquid Chromatography-Mass Spectrometry

Compared with LC, LC-MS shows higher sensitivity, higher specificity, with simpler sample preparation procedures. LC-MS has been widely used in the determination and analysis of solanesol. Zhao et al. [67] utilized liquid chromatography-tandem mass spectrometry (LC-MS/MS) for the determination of free solanesol in tobacco: the sample was prepared by ultrasonic extraction with methanol, liquid-liquid extraction, concentration, reconstitution, and filtration, followed by separation with the Symmetry Shield™ RP18 column. A mixture of acetonitrile-isopropanol (50:50, V/V) containing 2 mM ammonium acetate was used as mobile phase, and the determination was performed in positive-ion mode. The standard addition method was used for quantitative analysis. This method had a limit of quantification of 5.0 ng/mL, an intraday and interday precision of 0.89% and 1.12%, respectively, and a recovery of 97.72%–99.67%. Zhou and Lin [68] utilized liquid chromatography-electrospray ionization single stage quadrupole mass spectrometry (LC-ESI-MS) to quantify the total solanesol in tobacco: the Hypersil ODS column was used as the chromatography column and a mixture of methanol-isopropanol (40:60, *V*/*V*) was used as mobile phase. The determination was performed with isocratic elution in positive-ion mode. The minimum detection limit of this method was 0.12 ng, and the average recovery was 99.10%. Chen et al. [69] established a liquid chromatography-atmospheric pressure chemical ionization single stage four-stage mass spectrometry (LC-APCI-MS) method for the determination of total solanesol in tobacco: the chromatography column used was the Alltima C8 column (250 × 4.6 mm), the mobile phase was a methanol-water (98:2, V/V) mixture, and the flow rate was 0.8 mL/min. Meanwhile, the effects of LC-APCI-MS, LC-ESI-MS, and LC-UV on the determination of solanesol in tobacco were compared. LC-APCI-MS was more sensitive than LC-ESI-MS and LC-UV, but LC-UV displayed better precision and reproducibility.

### 6.3. Gas Chromatography

The boiling point of solanesol is 685.6 °C, which imposes many difficulties during separation by GC. However, domestic and foreign researchers still conducted research on solanesol determination using GC. Severson et al. [70] utilized hexane as extractant, saponification as pretreatment, Dexsil 300 as chromatography column, myristic glycerol diester as internal standard, N, O-bistrimethylsilyl acetamide (BSA) as the derivatization reagent, and used gas chromatography thermal conductivity detector (GC-TCD) to determine the content of free solanesol and total solanesol in tobacco leaves. Sheen et al. [71] developed a gas chromatography flame ionization detector (GC-FID) to determine the total solanesol content in tobacco leaves: the following treatments such as column chromatography, concentration, and saponification, the sample was separated in a Gas-Chrom Q (80-100 mesh) glass column coated with 1% OV-101, and cholesteryl laurate was used as an internal standard for quantification. Chaberlain et al. [72] quantified the total solanesol in tobacco leaves using capillary gas chromatography: following treatments such as saponification, thermal reflux, liquid-liquid extraction, and concentration, BSA was added for derivatization. The sample was then separated in the SE-54 capillary column. FID was used for detection and butyl tridecanoate was used as an internal standard for quantification. Liu et al. [73] used GC-FID to determine the content of solanesol and other substances in tobacco leaves: after saponification, the substances in tobacco leaves, such as solanesol, were extracted by ultrasonic extraction, and then bis (trimethylsilyl) trifluoroacetamide and trimethylchlorosilane were added for the derivatization reaction. Separation was performed in a DB-5 column, and quantitative analysis was performed using 5α-cholestane as an internal standard. This method had a recovery of 85%–105% and a relative standard deviation (RSD) of less than 4%.

### 6.4. Thin Layer Chromatography

TLC is characterized by its ease of operation, simple equipment requirement, fast speed, and low cost. Woollen and Jones [74] performed quantitative analysis of solanesol in tobacco leaves using silica gel G as the stationary phase, benzene as the developing reagent, phosphomolybdic acid-ethanol (1.25%, *W*/*V*) as the indicator. The sample was baked at 120 °C for 20 min until the spots developed distinct color (solanesol spots were dark blue). The linear range of this method was 0.25–2.5 μg of solanesol, with an RSD of less than 3%. Li et al. [75] determined the solanesol content in tobacco leaves using a silica gel G high-efficiency thin-layer dual-wavelength scanning method (the measurement wavelength was 540 nm, and the reference wavelength was 700 nm): hexane-ethyl acetate-acetic acid (4:1:0.5, *V*/*V*/*V*) was used as the developing reagent, vanillin-concentrated sulfuric acid solution was used as the chromogenic reagent, and the sample was baked at 100 °C for 5 min until the spots developed distinct color (solanesol spots were purple). This method had an average recovery of 95.6% and an RSD of less than 5%. Yu et al. [76] determined the solanesol content in tobacco leaves using TLC single-wavelength (620 nm) scanning: the developing reagent was petroleum ether-ethyl acetate (4:1, *V*/*V*) and the chromogenic reagent was vanillin sulfuric acid-ethanol solution. Furthermore, the sample was developed at 100 °C for 5 min, and the solanesol spots appeared purple. This method was feasible, with high accuracy, good stability, and high recovery.

### 6.5. Near Infrared Diffuse Reflectance Spectrophotometry

NIRDRS exhibits advantages such as rapid detection, simple sample preparation, low cost, no required reagents, no pollution, and easy on-line detection. This method has become an analytical tool that has attracted much attention in recent years. Fu et al. [77] utilized LC-UV to determine the chemical composition values of free solanesol in tobacco, and then used NIRDRS combined with least squares regression to perform first-derivative spectral pre-processing. They established a correction model for the free solanesol in tobacco in the near-infrared spectral region of 7836.02–3893.62 cm^−1^. The mean squared error by in-model cross validation was 0.108, the mean squared error of the prediction by external validation was 0.0822, and the average relative deviation was 5.3%. The predicted values were consistent with the chemical composition values.

### 6.6. Coulometric Titration

Coulometric titration is also used by many researchers to determine solanesol content because of its advantages such as ease of operation, low cost, no required standard materials, and accurate analytical results. Liu et al. [78] used a solution mixture consisting of a considerable molar concentration of potassium bromide and glacial acetic acid as the electrolyte and selected an appropriate current to perform electrolysis. The endpoint of the titration was indicated by applying a current between a double platinum electrode, and the solanesol content in the tobacco was calculated according to Faraday’s law. Zhao et al. [79] modified the above method by coating the double platinum electrode with perfluorosulfonic acid for use as an indicator, which greatly improved the precision of the method. Zheng [80] conducted coulometric titration of solanesol in tobacco leaves using a glacial acetic acid-methanol-water (72:8:20, *V*/*V*/*V*) solution containing 3.5 g of potassium bromide as solvent and at a constant current of 5 mA. Their experiments showed that data reproducibility is the best in the temperature range of 20–30 °C.

### 6.7. Indirect Iodometry

Indirect iodometry is a classic chemical analytical method that is broadly used for its high sensitivity, ease of operation, and low cost. Wei and Liu [81] used glacial acetic acid to establish an acidic reaction system and utilized the Br_2_ generated quantitatively from the KBrO_3_-KBr reaction to perform an addition reaction with solanesol. The remaining Br_2_ was determined using the iodometric method, and the solanesol content in tobacco leaves was then indirectly calculated. This method had a spiked recovery of 96%–101% and an RSD of 1.36%.

## 7. Conclusions and Future Perspectives

Due to the presence of several nonconjugated double bonds, solanesol possesses strong free radical absorption ability and antioxidant activity. Notably, the anti-inflammatory, neuroprotective, and antimicrobial activities of solanesol have also been proven in several studies. Solanesol serves as a key intermediate in the synthesis of ubiquinone drugs, including coenzyme Q10 and vitamin K2, and the anticancer agent synergizer SDB. Other applications of solanesol include solanesol derivative micelles for the delivery of hydrophobic drugs, solanesol-derived scaffolds for the multimerization of bioactive peptides, and solanesol-anchored DNA for mediating vesicle fusion. Although significant progress has been made in research on the bioactivities and medicinal value of solanesol, few studies on the pharmacokinetics of solanesol and its derivatives have been performed. Therefore, more emphasis should be placed on research on drug development and pharmacokinetics of solanesol and its derivatives, to progressively elucidate their patterns of absorption, distribution, metabolism, and excretion within organisms.

Besides genetic factors, biotic stresses such as pathogen infections and environmental factors such as temperature, illumination, and agronomic measures influence solanesol accumulation in plants. Notably, moderately high temperatures can promote solanesol accumulation through an increase in the net photosynthesis rate of tobacco leaves and the expression of key enzyme genes related to solanesol synthesis. Extraction technology of solanesol primarily includes ammonia leaching pretreatment assisted extraction, dynamic saponification extraction, ultrasonic assisted extraction, molecular distillation extraction, supercritical fluid extraction, bio-enzymatic extraction, and MIPs extraction. Determination methods of solanesol primarily include LC, LC-MS, GC, TLC, NIRDRS, coulometric titration, and indirect iodometry. During tobacco harvesting, tobacco leaves that do not meet harvesting standards because of factors such as planting techniques and climate are left on the tobacco plants or discarded. Fragments that cannot be used in tobacco products are also produced during tobacco leaf processing. Open-air storage or incineration of these tobacco residues will result in resource wastage and environmental pollution. In China, the medicinal value of tobacco has been well-documented in Chinese *materia medica* and traditional Chinese medical literature. If bioactive compounds such as solanesol [1,2,3,4] and cembranoids [82,83,84,85] in tobacco residues can be extracted, refined, and used in industries such as plant medicines, cosmetics, and functional foods, disposal and utilization of the residues can be achieved at the same time. The extraction of multiple chemical components with utilization value from tobacco for application in the medical industry not only creates effective utilization routes for tobacco but also provides effective routes for the search of new drug sources.

## References

[1] Yan N., Liu Y., Gong D., Du Y., Zhang H., Zhang Z. (2015). Solanesol: A review of its resources, derivatives, bioactivities, medicinal applications, and biosynthesis. Phytochem. Rev..

[2] Yan N., Zhang H., Zhang Z., Shi J., Timko M.P., Du Y., Liu X., Liu Y. (2016). Organ-and growing stage-specific expression of solanesol biosynthesis genes in *Nicotiana tabacum* reveals their association with solanesol content. Molecules.

[3] Yan N., Liu Y., Zhang H., Du Y., Liu X., Zhang Z. (2017). Solanesol biosynthesis in plants. Molecules.

[4] Yan N., Du Y., Zhang H., Zhang Z., Liu X., Shi J., Liu Y. (2018). RNA sequencing provides insights into the regulation of solanesol biosynthesis in *Nicotiana tabacum* induced by moderately high temperature. Biomolecules.

[5] Arab M., Bahramian B., Schindeler A., Fathi A., Valtchev P., McConchie R., Dehghani F. (2019). A benign process for the recovery of solanesol from tomato leaf waste. Heliyon.

[6] Roe S.J., Oldfield M.F., Geach N., Baxter A. (2013). A convergent stereocontrolled synthesis of [3-^14^C] solanesol. J. Label. Compound. Radiopharm..

[7] Yan N., Zhao T., Xiang D., Gong D., Zhang H., Du Y., Liu X., Zhang Z., Liu Y. (2016). Cloning and expression analysis of solanesyl diphosphate synthase (*NtSPS*) genes in *Nicotiana tabacum*. Chin. Tob. Sci..

[8] Taylor M.A., Fraser P.D. (2011). Solanesol: Added value from Solanaceous waste. Phytochemistry.

[9] Campbell R., Freitag S., Bryan G.J., Stewart D., Taylor M.A. (2016). Environmental and genetic factors associated with solanesol accumulation in potato leaves. Front. Plant Sci..

[10] Xiang D., Yao Z., Liu Y., Gai X., Du Y., Zhang Z., Yan N., Wang A., Fu Q. (2017). Analysis on solanesol content and genetic diversity of Chinese flue-cured tobacco (*Nicotiana tabacum* L.). Crop Sci..

[11] Rowland R.L., Latimer P.H., Giles J.A. (1956). Flue-cured tobacco. I. Isolation of solanesol, an unsaturated alcohol. J. Am. Chem. Soc..

[12] Huang W., Li Z., Niu H., Wang J., Qin Y. (2008). Bioactivity of solanesol extracted from tobacco leaves with carbon dioxide–ethanol fluids. Biochem. Eng. J..

[13] Yao X., Bai Q., Yan D., Li G., Lü C., Xu H. (2015). Solanesol protects human hepatic L02 cells from ethanol-induced oxidative injury via upregulation of HO-1 and Hsp70. Toxicol. In Vitro.

[14] Yao X., Lu B., Lü C., Bai Q., Yan D., Wu Y., Hong Z., Xu H. (2017). Solanesol induces the expression of heme oxygenase-1 via p38 and Akt and suppresses the production of proinflammatory cytokines in RAW264.7 cells. Food Funct..

[15] Mehan S., Rajput M., Dudi R., Ghimire K. (2018). Neuroprotective strategies of solanesol in mitochondrial impairment in experimentally induced Huntington disease. J. Pharm. Toxicol..

[16] Li J., Chase H.A. (2010). Development of adsorptive (non-ionic) macroporous resins and their uses in the purification of pharmacologically-active natural products from plant sources. Nat. Prod. Rep..

[17] Qin B., Liu L., Wu X., Liang F., Hou T., Pan Y., Song S. (2017). mPEGylated solanesol micelles as redox-responsive nanocarriers with synergistic anticancer effect. Acta Biomater..

[18] Qin B., Liu L., Pan Y., Zhu Y., Wu X., Song S., Han G. (2017). PEGylated solanesol for oral delivery of coenzyme Q_10_. J. Agric. Food Chem..

[19] Ma Y., Hou T., Zhang J., Zhou Y., Xu R., Liu Y., Wang Y. (2011). Solanesol antioxidation. Food Res. Dev..

[20] Bai Q., Yu J., Su M., Bai R., Katumata G., Katumata M., Chen X. (2014). Antioxidant function of solanesol and its inhibitory effect on tyrosinase. J. Biomed. Eng..

[21] Paine A., Eiz-Vesper B., Blasczyk R., Immenschuh S. (2010). Signaling to heme oxygenase-1 and its anti-inflammatory therapeutic potential. Biochem. Pharmacol..

[22] Mayer M.P. (2013). Hsp70 chaperone dynamics and molecular mechanism. Trends Biochem. Sci..

[23] Banožić M., Banjari I., Jakovljević M., Šubarić D., Tomas S., Babić J., Jokić S. (2019). Optimization of ultrasound-assisted extraction of some bioactive compounds from tobacco waste. Molecules.

[24] Sridevi P., Vijayanand P., Raju M.B. (2017). Formulation and evaluation of anti-inflammatory herbal gel containing isolated solanesol. Ann. Phytomed..

[25] Zhang R., Wang X., Ren X., Li Q., Tang J., Tang L., Hua Y., Liu J., Wang H. (2018). The effect of solanesol on experimental models of periodontitis in rats. Chin. J. Ethnomed. Ethnopharm..

[26] Gao Y., Zhou X., Wang F., Zhang H. (2011). Interaction between solanesol and bovine serum albumin. Chem. Ind. For. Prod..

[27] Zheng Z., Diamond M.I. (2012). Huntington disease and the huntingtin protein. Prog. Mol. Biol. Transl. Sci..

[28] Mehan S., Monga V., Rani M., Dudi R., Ghimire K. (2018). Neuroprotective effect of solanesol against 3-nitropropionic acid-induced Huntington’s disease-like behavioral, biochemical, and cellular alterations: Restoration of coenzyme-Q10-mediated mitochondrial dysfunction. Indian J. Pharmacol..

[29] Chen H.B., Zhang J., Yu H.X., Hu Q.L. (2007). In vitro study on the antibacterial of a medicinal intermediate, solanesol. Qilu Pharm. Aff..

[30] Duan S., Du Y., Hou X., Li D., Ren X., Dong W., Zhao W., Zhang Z. (2015). Inhibitory effects of tobacco extracts on eleven phytopathogenic fungi. Nat. Prod. Res. Dev..

[31] Bajda A., Konopka-Postupolska D., Krzymowska M., Hennig J., Skorupinska-Tudek K., Surmacz L., Wojcik J., Matysiak Z., Chojnacki T., Skorzynska-Polit E. (2009). Role of polyisoprenoids in tobacco resistance against biotic stresses. Physiol. Plant..

[32] Bentinger M., Tekle M., Dallner G. (2010). Coenzyme Q-Biosynthesis and functions. Biochem. Biophys. Res. Commun..

[33] Sarmiento A., Diaz-Castro J., Pulido-Moran M., Kajarabille N., Guisado R., Ochoa J.J. (2016). Coenzyme Q_10_ supplementation and exercise in healthy humans: A systematic review. Curr. Drug Metab..

[34] Mezawa M., Takemoto M., Onishi S., Ishibashi R., Ishikawa T., Yamaga M., Fujimoto M., Okabe E., He P., Kobayashi K. (2012). The reduced form of coenzyme Q_10_ improves glycemic control in patients with type 2 diabetes: An open label pilot study. BioFactors.

[35] Hamidi M.S., Gajic-Veljanoski O., Cheung A.M. (2013). Vitamin K and bone health. J. Clin. Densitom..

[36] Villa J.K.D., Diaz M.A.N., Pizziolo V.R., Martino H.S.D. (2017). Effect of vitamin K in bone metabolism and vascular calcification: A review of mechanisms of action and evidences. Crit. Rev. Food Sci..

[37] Enokida H., Gotanda T., Oku S., Imazono Y., Kubo H., Hanada T., Suzuki S., Inomata K., Kishiye T., Tahara Y. (2002). Reversal of P-glycoprotein-mediated paclitaxel resistance by new synthetic isoprenoids in human bladder cancer cell line. Jpn. J. Cancer Res..

[38] Sidorova T.A., Nigmatov A.G., Kakpakova E.S., Stavrovskaya A.A., Gerassimova G.K., Shtil A.A., Serebryakov E.P. (2002). Effects of isoprenoid analogues of SDB-ethylenediamine on multidrug resistant tumour cells alone and in combination with chemotherapeutic drugs. J. Med. Chem..

[39] Xiao K., Dai Y., Shi W., Li J., Li Y., Yin S. (2012). Synthesis and antitumor activities of the diacid solanesyl 5-fluorouracil esters derivatives. Chin. J. Org. Chem..

[40] Daneial B., Joseph J.P.V., Ramakrishna G. (2017). Molecular dynamics simulation analysis of Focal Adhesive Kinase (FAK) docked with solanesol as an anti-cancer agent. Bioinformation.

[41] Srivastava S., Raj K., Khare P., Bhaduri A.P., Chander R., Raghubir R., Mahendra K., Rao C.V.N., Prabhu S.R. (2009). Novel hybrid natural products derived from solanesol as wound healing agents. Indian J. Chem..

[42] Alleti R., Rao V., Xu L., Gillies R.J., Mash E.A. (2010). A solanesol-derived scaffold for multimerization of bioactive peptides. J. Org. Chem..

[43] Flavier K.M., Boxer S.G. (2017). Vesicle fusion mediated by solanesol-anchored DNA. Biophys. J..

[44] Gai X., Liu Y., Yao Z., Du Y., Yan N., Zhang H., Dai P. (2017). Study on the correlation between solanesol accumulation and expression of gene encoding terpenoid synthetic enzymes in tobacco. Chin. Tob. Sci..

[45] Xiang D., Zhao T., Du Y., Zhang Z., Yan N., Huang W., Wang A., Fu Q., Gong Y., Liu Y. (2015). Genetic analysis on solanesol content of tobacco. Chin. Tob. Sci..

[46] Qiao R., Xia L., Liu L., Yue C. (2011). Effect of illumination intensity on solanesol content in tobacco. Guizhou Agric. Sci..

[47] Schepartz A.I., Ellington J.J., Burk L.G. (1978). Catalase activity and lipid contents of leaves from normal and sterile-flowered tobacco plants. Phytochemistry.

[48] Burton H.R., Leggett E., Philips R.E. (1989). Factors influencing the concentration of solanesol in burley tobacco. Beit. Tabakforsch. Int..

[49] Sun Y., Huang R., Xing X., Qi W., Su R., He Z. (2013). Enhanced extraction of solanesol from tobacco leaves by a new ammonia leaching pretreatment method. Fine Chem..

[50] Zhao C., Yang L., Zu Y., Xia L., Xiao C. (2007). Extraction of solanesol by dynamic saponification method. Chem. Eng..

[51] Zhang Z., Feng X. (2007). Study on ultrasonic assisted extraction of solanesol from tobacco leaves. J. Food Sci. Biotechnol..

[52] Qian C., Zhang J. (2009). The study of refining solanesol by molecular distillation. J. Chem. Eng. Chin. Univ..

[53] Wei H., Mi H., Liu Z. (2005). Extraction and separation of solanesol from *Nicotiana tobacum* by supercritical fluid extraction and silica gel column chromatography. Chin. Tradit. Herb. Drugs.

[54] Wang Y., Gu W. (2018). Study on supercritical fluid extraction of solanesol from industrial tobacco waste. J. Supercrit. Fluids.

[55] Wang X., Zhang Y., Zhang G., Yin Z. (2013). Improved extraction of solanesol from tobacco waste by enzymatic cell wall breaking. Chin. J. Biotechnol..

[56] Long J.P., Chen Z.B., Liu X.J., Du X.Y. (2015). Preparation and adsorption property of solanesol molecular imprinted polymers. Des. Monomers Polym..

[57] Ma X., Meng Z., Qiu L., Chen J., Guo Y., Yi D., Ji T., Jia H., Xue M. (2016). Solanesol extraction from tobacco leaves by Flash chromatography based on molecularly imprinted polymers. J. Chromatogr. B.

[58] Duan C., Chen Z., Liu X., Li K., Wang X., Jia W., Tang Z., Ruso J.M., Liu Z. (2019). Noble surface molecularly imprinted polymer modified titanium dioxide toward solanesol adsorption selectivity study. J. Mater. Res..

[59] Liu C., Zhang H., Du Y., Hou X., Li D., Yan N. (2015). The simultaneous extraction and saponification of tobacco solanesol using ultra performance liquid chromatography. Chin. Tob. Sci..

[60] Zhao Y., Du Q. (2007). Separation of solanesol in tobacco leaves extract by slow rotary counter-current chromatography using a novel non-aqueous two-phase solvent system. J. Chromatogr. A.

[61] Rao R.N., Talluri M.K., Krishna T.M., Ravindranath K. (2008). Continuous counter current extraction, isolation and determination of solanesol in *Nicotiana tobacum* L. by non-aqueous reversed phase high performance liquid chromatography. J. Pharmaceut. Biomed..

[62] Fan Z., Chen F., Zhou Y., Chen Z., Li X. (2015). Research progress of determination methods of solanesol in tobacco. Chin. Agric. Sci. Bull..

[63] Zhao J., Wang C., Sun X. (1997). Determination of solanesol in the extracts of tobacco leaves by high performance liquid chromatography (HPLC). Chin. J. Chromatogr..

[64] Zhang H., Wang H., Chen S. (2005). Extraction and determination of the solanesol in discarded tobacco leaf. J. Henan Univ. Technol..

[65] Li L., Qian Q. (2002). Determination of solanesol in *Nicotiana tobacum* L. by HPLC-ELSD. Chin. J. Pharm. Anal..

[66] Zhou H., Liu C. (2006). Rapid determination of solanesol in tobacco by high-performance liquid chromatography with evaporative light scattering detection following microwave-assisted extraction. J. Chromatogr. B.

[67] Zhao C., Li C., Zu Y. (2007). Rapid and quantitative determination of solanesol in *Nicotiana tabacum* by liquid chromatography–tandem mass spectrometry. J. Pharmaceut. Biomed..

[68] Zhou Y., Lin R. (2008). Determination of solanesol in the extracts of tobacco leaves by electrospray ionization mass spectrometry. J. Jiangsu Polytech. Univ..

[69] Chen J., Liu J., Lee F.S.C., Wang X. (2008). Optimization of HPLC-APCI-MS conditions for the qualitative and quantitative determination of total solanesol in tobacco leaves. J. Sep. Sci..

[70] Severson R.F., Ellington J.J., Schlotzhauer P.F., Arrendale R.F., Schepartz A.I. (1977). Gas chromatographic method for the determination of free and total solanesol in tobacco. J. Chromatogr. A.

[71] Sheen S.J., Davis D.L., DeJong D.W., Chaplin J.F. (1978). Gas-liquid chromatographic quantification of solanesol in chlorophyll mutants of tobacco. J. Agric. Food Chem..

[72] Chaberlain W.J., Severson R.F., Chortyk O.T., Sisson V.E. (1990). Determination of solanesol in tobacco by capillary gas chromatography. J. Chromatogr. A.

[73] Liu Y., Yong G., Xu Y., Zhu D., Tong H., Liu S. (2010). Simultaneous determination of free and esterified fatty alcohols, phytosterols and solanesol in tobacco leaves by GC. Chromatographia.

[74] Woollen B.H., Jones D.H. (1971). Analytical methods for tobacco lipids: 1. A rapid method for the estimation of solanesol by thin-layer densitometry. J. Chromatogr. A.

[75] Li L., Ma Z., Qian Q., Cong X. (2002). Determination of solanesol in tobacco leaves by TLC scanning. Chin. Tradit. Herb. Drugs.

[76] Yu H., You W., Rao L., Cao Y., Li G., Zhang A. (2006). Detecting content of solanesol in tobacco leaves by TLC. Sichuan Food Ferment..

[77] Fu Q., Du Y., Zhang H., Hou X., Li Y., Li D. (2015). Determination of solanesol content of flue-cured tobacco leaves by near-infrared diffuse reflectance technology. Nat. Prod. Res. Dev..

[78] Liu K., Liu M., Li D. (1998). Determination of solanesol by coulometric titration. Anal. Lett..

[79] Zhao G., Qu J., Liu M., Liu K., Du Z. (2002). Application of chemical modified electrode in coulometric titration for determination of solanesol. Anal. Lett..

[80] Zheng K. (2003). One kind of method separating purification and measuring solanesol content from matter distilled of tobacco leaf. J. Guizhou Norm. Univ..

[81] Wei Y., Liu J. (2009). Test solanesol with indirect iodimetry. Appl. Chem. Ind..

[82] Duan S., Du Y., Hou X., Yan N., Dong W., Mao X., Zhang Z. (2016). Chemical basis of the fungicidal activity of tobacco extracts against *Valsa mali*. Molecules.

[83] Yan N., Du Y., Liu X., Zhang H., Liu Y., Zhang P., Gong D., Zhang Z. (2016). Chemical structures, biosynthesis, bioactivities, biocatalysis and semisynthesis of tobacco cembranoids: An overview. Ind. Crops Prod..

[84] Yan N., Du Y., Liu X., Zhang H., Liu Y., Shi J., Xue S.J., Zhang Z. (2017). Analyses of effects of α-cembratrien-diol on cell morphology and transcriptome of *Valsa mali* var. *mali*. Food Chem..

[85] Yan N., Du Y., Liu X., Zhang H., Liu Y., Zhang Z. (2019). A review on bioactivities of tobacco cembranoid diterpenes. Biomolecules.

